# Investigating protease-mediated peptides of inflammation and tissue remodeling as biomarkers associated with flares in psoriatic arthritis

**DOI:** 10.1186/s13075-024-03332-7

**Published:** 2024-05-27

**Authors:** Solveig Skovlund Groen, Signe Holm Nielsen, Anne Christine Bay-Jensen, Mozhgan Rasti, Darshini Ganatra, Katerina Oikonomopoulou, Vinod Chandran

**Affiliations:** 1 ImmunoScience, Biomarkers and Research, Nordic Bioscience, Herlev, Denmark; 2 Biomedical Sciences, University of Copenhagen, Copenhagen N, Denmark; 3 Biotechnology and Biomedicine, Technical University of Denmark, Kgs. Lyngby, Denmark; 4 Schroeder Arthritis Institute, Krembil Research Institute, University Health Network, Toronto, ON Canada; 5 Department of Medicine, Division of Rheumatology, Department of Laboratory Medicine and Pathobiology, Institute of Medical Science, University of Toronto, Toronto, ON Canada

**Keywords:** Psoriatic arthritis, Extracellular matrix, Biomarker, Tissue remodeling, Flares

## Abstract

**Background:**

Psoriatic arthritis (PsA) is an inflammatory arthritis associated with psoriasis. PsA disease involves flares, which are associated with increased joint inflammation and tissue remodeling. There is a need for identifying biomarkers related to PsA disease activity and flares to improve the management of PsA patients and decrease flares. The tissue turnover imbalance that occurs during the inflammatory and fibro-proliferative processes during flares leads to an increased degradation and/or reorganization of the extracellular matrix (ECM), where increased proteolysis plays a key role. Hence, protease-mediated fragments of inflammatory and tissue-remodeling components could be used as markers reflecting flares in PsA patients.

**Methods:**

A broad panel of protease-mediated biomarkers reflecting inflammation and tissue remodeling was measured in serum and synovial fluid (SF) obtained from PsA patients experiencing flares (acutely swollen joint[s], PsA-flare). In serum, biomarker levels assessed in PsA-flare patients were compared to controls and in early-diagnosed PsA patients not experiencing flares (referred to as PsA without flare). Furthermore, the biomarker levels assessed in SF from PsA-flare patients were compared to the levels in SF of osteoarthritis (OA) patients.

**Results:**

In serum, levels of the PRO-C3 and C3M, reflecting formation and degradation of the interstitial matrix, were found significantly elevated in PsA-flare compared to controls and PsA without flare. The remodeling marker of the basement membrane, PRO-C4, was significantly elevated in PsA-flare compared to PsA without flare. The inflammation and immune cell activity related markers, CRPM, VICM, and CPa9-HNE were significantly elevated in PsA-flare patients compared to controls and PsA without flare. In addition, VICM (AUC = 0.71), CPa9-HNE (AUC = 0.89), CRPM (AUC = 0.76), and PRO-C3 (AUC = 0.86) showed good discriminatory performance for separating PsA-flare from PsA without flare. In SF, the macrophage activity marker, VICM, was significantly elevated whereas the type II collagen formation marker, PRO-C2, was significantly reduced in the PsA-flare compared to OA. The combination of five serum markers reflecting type III and IV collagen degradation (C3M and C4M, respectively), type III and VI collagen formation (PRO-C3 and PRO-C6, respectively), and neutrophil activity (CPa9-HNE) showed an excellent discriminatory performance (AUC = 0.98) for separating PsA-flare from PsA without flares.

**Conclusions:**

The serum biomarker panel of C3M, C4M, PRO-C3, PRO-C6, and CPa9-HNE reflecting synovitis, enthesitis, and neutrophil activity may serve as novel tool for quantitatively monitoring flares in PsA patients.

**Supplementary Information:**

The online version contains supplementary material available at 10.1186/s13075-024-03332-7.

## Introduction

PsA is a chronic, immune-mediated, inflammatory musculoskeletal disease that affects a third of the patients with psoriasis [1]. Because of its progressive nature, insufficient treatment of PsA can cause disability with permanent joint damage within the first few years of disease. Therefore, early diagnosis as well as proper management of PsA patients is crucial to improve the care and quality of life for these patients [2]. However, early diagnosis, disease activity assessment, and prediction of outcome in PsA is challenging, especially since it is a heterogeneous disease [3]. PsA patients can present with various clinical manifestations, such as psoriasis (skin and nail), synovitis, enthesitis, dactylitis, and spondylitis [4].

Biomarkers have valuable applications in detection of disease and monitoring health status, including diagnosing, staging, predicting progression of disease, and response to therapy. However, unlike rheumatoid arthritis and systemic lupus erythematosus, there are no biomarkers available for clinical use in PsA [5]. Recently, The TIght Control Of Psoriatic Arthritis (TICOPA) trial confirmed the benefit of regular disease activity assessment through a treat-to-target approach using objective outcome measures. Here, the regular disease activity assessment significantly improved joint outcomes for newly diagnosed patients, with no unexpected serious adverse events reported [6]. In this regard, biomarkers of disease activity could aid in the tight regular control of disease activity in PsA and help improve outcomes. A disease flare is defined as worsening of disease activity, particularly in duration and intensity. The occurrence of flares usually requires initiating or changing therapy. PsA flares have not been well characterized and currently no biomarkers for PsA flares are available [7].

Proteolysis is an important process during inflammation and has key roles during the progression of arthritis. Numerous biomarker candidates for assessing the degree of inflammation and disease activity can be identified among fragments generated in response to proteolytic enzyme activity at target tissues, including joints of PsA patients [8]. In PsA, the inflammatory load perturbs tissue homeostasis leading to altered balance of the proteolytic enzymes and ECM [9]. During this process, proteases, such as matrix metalloproteinases (MMPs), extensively cleave the ECM, and other inflammatory components, leading to unique protease-mediated breakdown products of collagens and pro-inflammatory effector molecules. These disease-specific protein fragments are released into the circulation and can be conveniently quantified in serum or SF as biomarkers linked to joint tissue remodeling and inflammation.

To meet the need for identifying reliable biomarkers of flares in PsA, it was hypothesized that protease-mediated fragments of inflammation and tissue remodeling could serve as markers of PsA patients experiencing flares. The presence of flares indicates elevated disease activity and manifests with increased levels of inflammation and tissue remodeling in the joints. The tissue turnover imbalance that arises during these inflammatory and fibro-proliferative processes leads to an increased degradation and/or reorganization of the ECM, where an elevated proteolytic load plays a key role. The serological biomarkers measured in this study are cleavage end products mediated by proteases that take part in these proteolytic processes and, therefore, these biomarkers can serve as potent candidates for assessing elevated disease activity in PsA patients undergoing flares. Additionally, the study aimed to evaluate these proteolytic markers in SF of PsA patients with flares, as another source of inflammatory markers in PsA in the immediate proximity of the inflamed joint where all the proteolytic events are primarily expected to take place.

## Methods

### Study population and design

Serum samples were obtained from 30 PsA patients with flares (PsA-flare) and analyzed together with 99 self-reported healthy donors (controls) and 98 early-diagnosed PsA patients not experiencing flares (PsA without flare). Patients with flares in this study were defined as those experiencing worsening of disease activity, particularly in duration and intensity presenting with at least one swollen and tender joint. Patients with flares required joint aspiration and intra-articular corticosteroid injection for acute onset pain and swelling in at least one joint, and on examination had at least 1 swollen and tender joint, typically the knee joint. These patients were diagnosed by a rheumatologist and satisfied Classification Criteria for Psoriatic Arthritis (CASPAR). PsA patients with no recent change in disease activity were categorized as PsA patients without flares (hereafter refer to as PsA without flare). These patients may have active disease but did not have a flare as defined above. 75% of PsA-flare and 20% of PsA without flare patients received conventional disease modifying anti-rheumatic drugs (c-DMARDs). 40% of the PsA-flare patients received targeted DMARDs (t-DMARDs) therapy, while the PsA without flares had never received t-DMARDs. The serum samples were stored in the biobank (-80 °C) until biomarker analysis. In addition, SF samples were obtained from 54 OA patients during total knee replacement surgery and compared to SF obtained during joint aspirations in an outpatient PsA clinic from 57 PsA-flare patients as defined above. The 57 SF samples from the PsA-flare patients included 30 samples matching the serum samples mentioned above plus 27 additional PsA-flare SF samples (without matching serum samples). The SF samples were centrifuged to remove cells and stored in the biobank (-80 °C). All patients were identified from cohorts followed prospectively from 2009 to 2020 at Toronto Western Hospital, Canada, after appropriate ethics approval.

### Biomarker quantification

PsA is a heterogeneous disease with local and systemic manifestations involving multiple tissues. Thus, several biomarkers reflecting synovitis, enthesitis, cartilage remodeling, immune cell activity, and systemic inflammation were measured in serum and SF samples. In the serum samples, seven markers related to ECM turnover and three markers related to inflammation were measured using ELISA-based technology, according to the manufacturer’s instructions (Nordic Bioscience A/S, Herlev, Denmark; Table 1): markers of type I, III, and IV, VI collagen degradation (nordicC1M™, cat no. 1000-01, nordicC3M™ cat no. 1200-02, nordicC4M™ cat no. 1300-02, nordicC6M™ cat no. 1500-01, respectively), markers of type III, IV, and VI collagen formation (nordicPRO-C3™ cat no. 1700-06, nordicPRO-C4™ cat no. 8000, nordicPRO-C6™ cat no. 4000-02, respectively), and markers of inflammation (nordicVICM™ cat no. 1800, nordicCPa9-HNE™ cat no. R1031-00, nordicCRPM™ cat no. 7000, respectively). Additionally, markers of type II collagen formation (nordicPRO-C2™ cat no. N120-00), aggrecan degradation (nordicARGS™ cat no. 4900), fibronectin remodeling (nordicFBN-C™, cat no. N101-00), inflammatory markers (nordicVICM™ cat no. 1800, nordicCPa9-HNE™ cat no. R1031-00, respectively) were measured in SF using assays manufactured by Nordic Bioscience, as previously described (Table 1). Each ELISA plate included samples from each of the different disease groups in duplicates. Markers in the samples were re-assayed if the coefficient of variation (CV) between the duplicates was higher than 20%.

**Table 1:** Overview of measured biomarkers representing inflammation and ECM turnover. MMP, matrix-metalloprotease; ADAMTS, a disintegrin and metalloproteinase with thrombospondin motifs; SF, synovial fluid

Biomarker	Description of measures	Serum	SF	Reference
Collagen degradation biomarkers				
C1M	MMP-2, -9, -13 mediated degradation of type I collagen	X		[22]
C3M	MMP-9 mediated degradation of type III collagen	X		[23]
C4M	MMP-2, -9, -12 mediated degradation of type IV collagen	X		[24]
C6M	MMP-2, -9 mediated degradation of type VI collagen	X		[25]
Collagen formation biomarkers				
PRO-C2	N-terminal propeptide of type II collagen (PIIBNP)		X	[26]
PRO-C3	N-terminal propeptide of type III collagen (PIIINP)	X		[27]
PRO-C4	Internal epitope in the 7 S domain of type IV collagen	X		[28]
PRO-C6	C-terminal NC domain of type VIa3 collagen (Endotrophin)	X		[29]
Cartilage remodeling biomarkers				
ARGS	ADAMTS-4 and − 5 mediated aggrecan degradation		X	[30]
FBN-C	C-terminal of fibronectin		X	[31]
Immunoinflammation-mediated biomarkers				
VICM	Citrullinated and MMP-2 degraded fragment of vimentin	X	X	[32]
CPa9-HNE	Human neutrophil elastase mediated fragment of the calprotectin subunit S100A9	X	X	[21]
CRPM	MMP-1 and − 8 mediated fragment of C-reactive protein	X		[33]

### Statistical analysis

The demographic summary statistics were calculated for each group with either mean ± standard deviation (SD), or median (interquartile range, IQR), or for categorical variables (presence/absence, yes/no) shown as presence or yes (%). Following tests were applied in the demographics table: Mann-Whitney for two groups of variables, Kruskal Wallis for variables with more than two groups, and Pearson’s chi-square for categorical data. To investigate the differences between the serum biomarker levels of controls, PsA without flare, and PsA-flare, presented by Tukey’s plot, the Kruskal-Wallis test followed by Dunn’s multiple comparison test was performed. For the comparison of the SF biomarker levels between OA and PsA-flare, presented by Tukey’s plot, the logistic regression model (adjusting for age) was applied. To describe the discrimination accuracy of each biomarker, as well as of a combination of the candidate markers between the disease groups, in serum and SF the receiver operating characteristic (ROC) curve analyses was performed and the area under the curve (AUC) was calculated. The Wald *p*-value and 95% confidence intervals were calculated for the multivariable analysis. Furthermore, associations between biomarker levels and clinical scores including demographics were evaluated using Spearman rank correlation. *p*-values below 0.05 were considered significant.

## Results

The demographics and clinical characteristics of the patients included in the study are summarized in Table 2.

**Table 2:** Demographics and clinical characteristics of controls, PsA without flare, and PsA-flare patients

**Serum**	**Controls** (***n* = 99**)	**PsA without flare** (***n* = 98**)	**PsA-flare** (***n* = 30**)	***p*** **-value**
Age, years^a^	45.9 (10.6)	45.9 (12.5)	45.9 (13.2)	1.000
Sex, Female^c^	44 (44%)	46 (47%)	8 (27%)	0.138
BMI (kg/m^2^)^a^	27.9 (5.8)	28.0 (6.1)	26.6 (3.6)	0.768
PsA duration (years)^a^	N/A	1.9 (3.4)	11.5 (10.0)	< 0.0001
PASI^a^	N/A	3.0 (4.2)	0.9 (2.4)	0.0008
Nail lesions^c^	N/A	45 (47%)	11 (40%)	0.373
SJC^b^	N/A	2 (0–23)	1 (1–6)	0.110
TJC^b^	N/A	4 (0–32)	1 (1–11)	0.0002
AJC^b^	N/A	5 (0–32)	1 (1–11)	< 0.0001
Enthesitis^c^	N/A	37 (38%)	5 (17%)	0.032
Dactylitis^c^	N/A	22 (24%)	0 (0%)	0.032
Axial involvement^c^	N/A	36 (40%)	5 (17%)	0.018
hsCRP^a^	N/A	6.5 (9.3)	5.6 (9.9)	0.676
DAPSA^a^	N/A	24.3 (15.1)	16.0 (13.5)	0.002
NSAID^c^	N/A	55 (56%)	12 (40%)	0.123
c-DMARD^c^	N/A	20 (20%)	21 (75%)	< 0.0001
t-DMARD^c^	N/A	0 (0%)	12 (40%)	< 0.0001

**Synovial fluid**	**OA** (***n* = 54**)	**PsA-flare** (***n* = 57**)	***p*** **-value**	
Age, years^a^	62.1 (12.0)	47.9 (13.7)	< 0.0001	
Sex, Female^c^	26 (48%)	20 (35%)	0.165	
c-DMARD^c^	N/A	21 (75%)	N/A	
t-DMARD^c^	N/A	30 (63%)	N/A	

^a^Mean ± SD, ^b^Median (interquartile range), ^c^Categorical data (presence/absence, yes/no) shown as presence or yes (%). PsA, psoriatic arthritis, OA, osteoarthritis; PASI, psoriasis area and severity index; SJC, Swollen joint count; TJC, Tender joint count; AJC, Active (swollen or tender) joint criteria; DAPSA, Disease activity in PsA; BMI, body mass index; t-DMARD, targeted disease-modifying anti-rheumatic drug; c-DMARD, conventional disease-modifying antirheumatic drug; hsCRP, high-sensitive c-reactive protein; N/A, not applicable

### Inflammation and extracellular matrix turnover

**Fig. 1:**
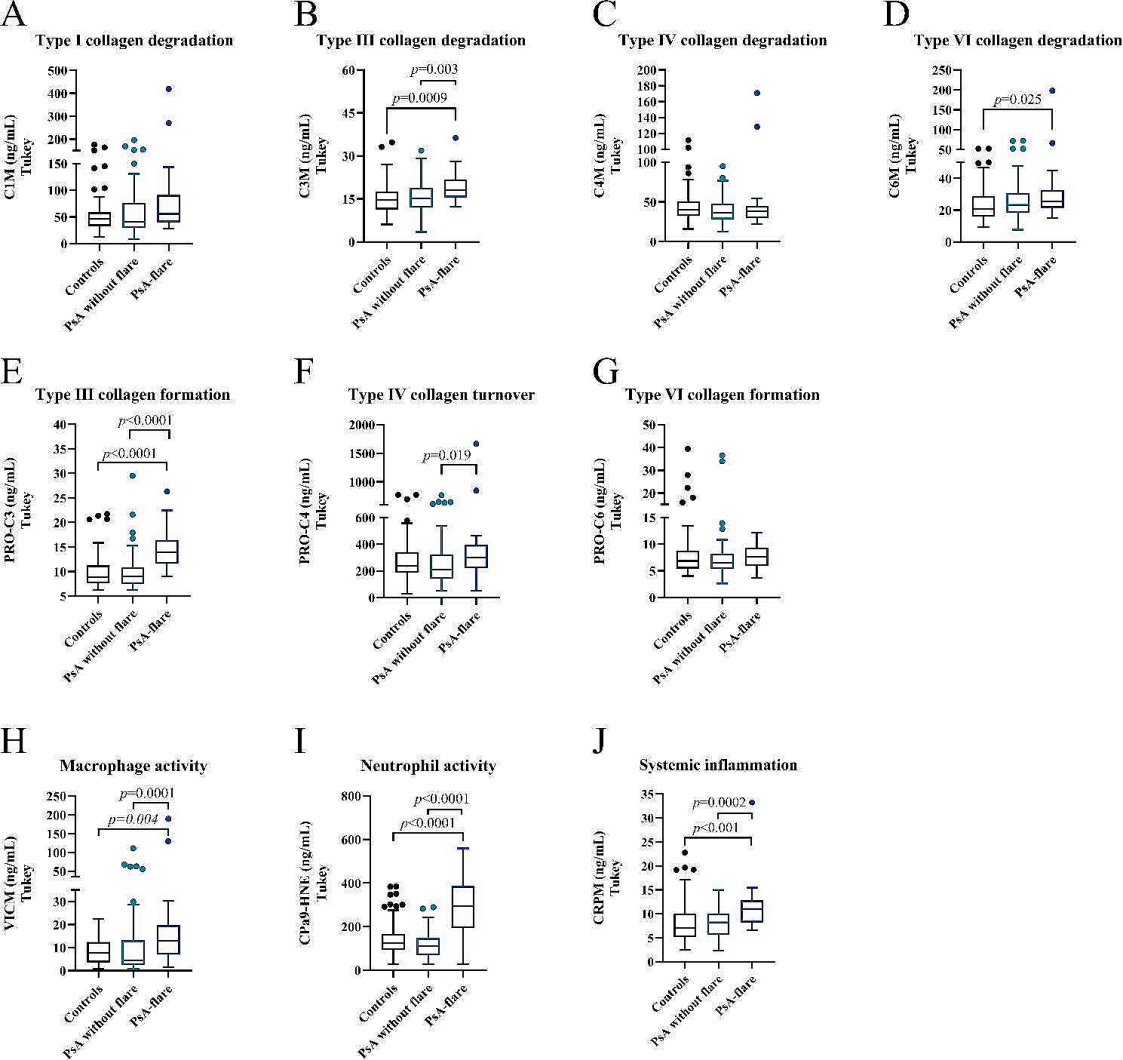
**A-J**. Biomarker quantification in serum from controls, PsA patients without flare (PsA), and PsA patients with flare (PsA-flare). ECM, extracellular matrix; PsA, Psoriatic Arthritis

Serum levels of collagen and inflammatory markers are presented in Fig. 1; Table 3. For the collagen biomarkers, levels of C1M and C4M, measuring type I and IV collagen degradation respectively, were not significantly altered between the different groups (Fig. 1A, C). However, significantly elevated levels of C3M and C6M, measuring type III and VI collagen degradation, were found between controls and PsA-flare (C3M: *p* = 0.0009, C6M: *p* = 0.025, Fig. 1B, D). In addition, C3M was significantly higher in PsA-flare compared to PsA without flare (*p* = 0.003, Fig. 1B). The fibrotic marker of type III formation, PRO-C3, demonstrated significantly elevated levels between the controls and PsA-flare, and between PsA-flare and PsA without flare (*p* < 0.0001, Fig. 1E). PRO-C4, measuring type IV collagen turnover was enhanced in the group with PsA-flare compared to PsA without flare (*p* = 0.019, Fig. 1F). No significant difference was seen between groups with PRO-C6, assessing type VI collagen formation (Fig. 1G). Regarding the inflammatory biomarkers indicating macrophage (VICM), and neutrophil (CPa9-HNE), and systemic inflammation (CRPM), all markers were highly elevated in PsA-flare compared to controls and PsA without flare groups (VICM: *p* = 0.004; *p* = 0.0001, CPa9-HNE: *p* < 0.0001, CRPM: *p* < 0.001; *p* = 0.0002, Fig. 1H, I, J). No significant differences were observed between the controls and PsA without flare patients for all the markers tested in serum.

**Table 3:** Serum biomarkers in controls, PsA without flare, and PsA-flare patients. The median (Interquartile Range 25–75%) is presented for controls, PsA without flare, and PsA-flare. PsA, psoriatic arthritis

Biomarker	Controls	PsA without flare	PsA-flare	*p*-value
C1M (ng/mL)	46.18 (33.21–58.74)	41.29 (29.33–76.32)	55.92 (40.21–91.08)	0.0573
C3M (ng/mL)	14.64 (11.38–17.69)	15.13 (12.02–18.89)	18.04 (15.54–18.04)	0.0013
C4M (ng/mL)	39.94 (32.49–50.92)	36.46 (28.01–47.81)	38.38 (29.35–44.82)	0.1741
C6M (ng/mL)	20.72 (15.83–28.92)	23.26 (18.29–30.58)	25.40 (21.40-32.61)	0.0198
PRO-C3 (ng/mL)	8.764 (7.6-11.26)	9.054 (7.46–10.81)	13.89 (11.58–16.36)	< 0.0001
PRO-C4 (ng/mL)	239.4 (186.9-340.8)	208.9 (140.8-323.6)	302.0 (220.7-395.2)	0.0244
PRO-C6 (ng/mL)	6.834 (5.14–8.80)	6.492 (5.13–8.17)	7.622 (5.90–9.36)	0.0992
VICM (ng/mL)	7.648 (3.61–12.44)	4.394 (2.53–13.13)	12.92 (6.88–19.77)	0.0001
CPa9-HNE (ng/mL)	125.0 (91.70-165.80)	110.0 (68.78–147.90)	294.4 (192.3-386.5)	< 0.0001
CRPM (ng/mL)	7.092 (5.18–10.06)	8.216 (5.63–10.06)	11.04 (8.19–12.78)	< 0.0001

**Fig. 2:**
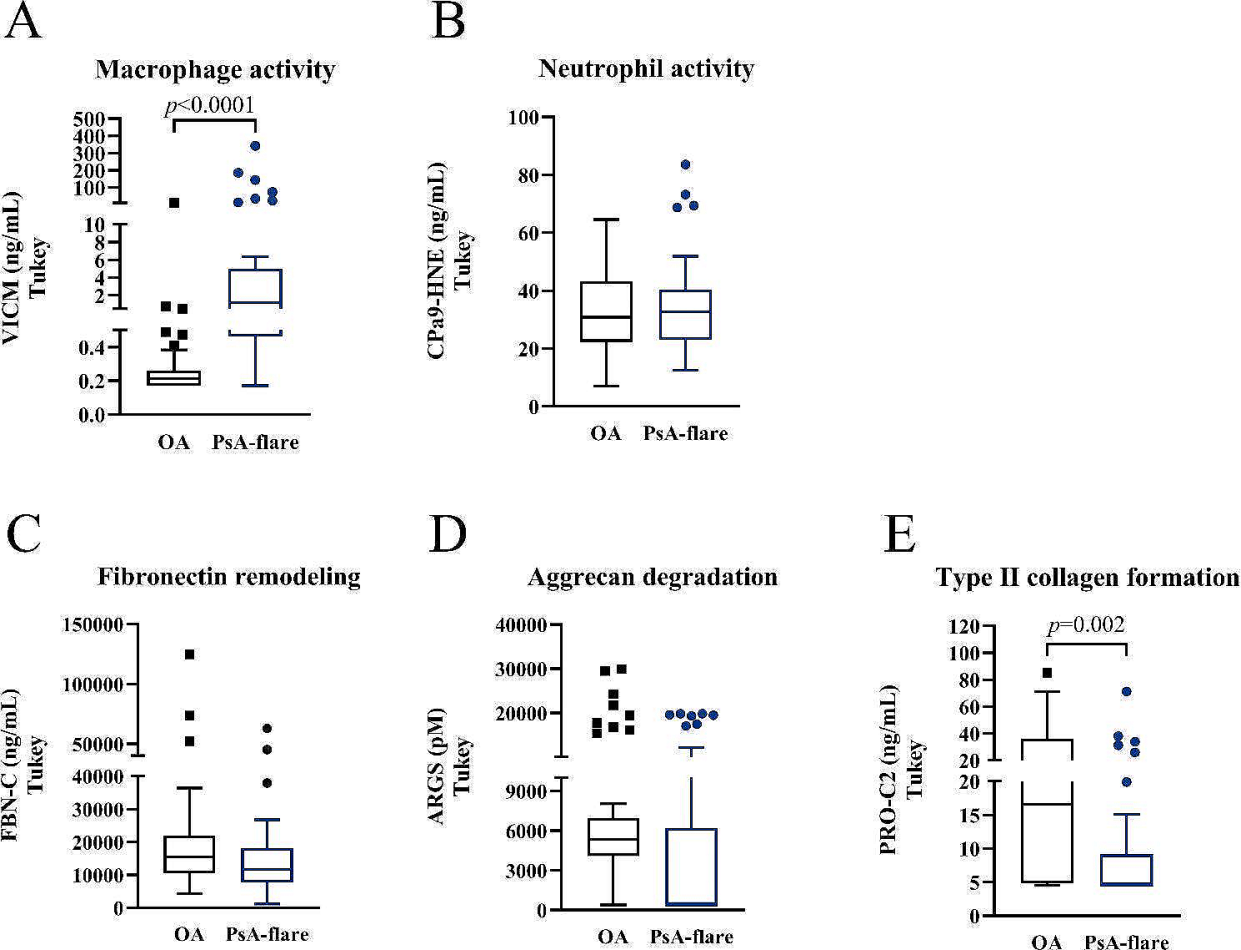
**A-E**. Biomarker quantification in synovial fluid from OA patients. PsA, Psoriatic Arthritis; OA, Osteoarthritis

In addition to the biomarker levels assessed in serum samples, levels of markers related to inflammation and cartilage remodeling were analyzed in SF samples from the PsA-flare patients as well as patients with OA, as a less inflammatory control. The macrophage activity marker, VICM, was clearly elevated in the PsA-flare patients compared to the OA patients (*p* < 0.0001, Fig. 2A), while the neutrophil activity marker was not significantly different (Fig. 2B). Of the markers reflecting cartilage remodeling, aggrecan degradation and fibronectin turnover, ARGS and FBN-C, levels were not significantly different between the OA and PsA-flare patients (Fig. 2C, D). Nevertheless, the type II collagen formation marker, PRO-C2, was significantly elevated in the OA patients compared to the PsA-flare (*p* = 0.002, Fig. 2E; Table 4).

**Table 4:** Biomarkers measured in synovial fluid from OA and PsA-flare patients. The median (Interquartile Range 25–75%) is presented for OA and PsA. OA, osteoarthritis; PsA, psoriatic arthritis

	OA	PsA-flare	*p*-value
VICM (ng/mL)	0.2120 (0.17–0.26)	1.196 (0.46–5.03)	< 0.0001
CPa9-HNE (ng/mL)	30.80 (22.25–43.35)	32.70 (23.05–40.35)	0.341
FBN-C (ng/mL)	15,539 (10,452–22,091)	11,662 (7646–18,185)	0.565
ARGS (pM)	5351 (4085–6958)	400 (400–6182)	0.067
PRO-C2 (ng/mL)	16.59 (4.9-36.18)	4.510 (4.51–9.19)	0.002

Thirty of the PsA-flare patients with SF samples had matching serum samples that were analyzed to investigate potential marker differences in the circulation versus the local joint microenvironment. Results from the analysis showed that the mean levels of CPa9-HNE and VICM were significantly elevated in serum compared to synovial fluid (Supplementary Fig. 1A-B). No correlation was observed between the systemic and local levels of the PsA-flare patients (Supplementary Table 1).

### Ability of biomarkers to discriminate between PsA-flare and PsA without flare patients

**Fig. 3:**
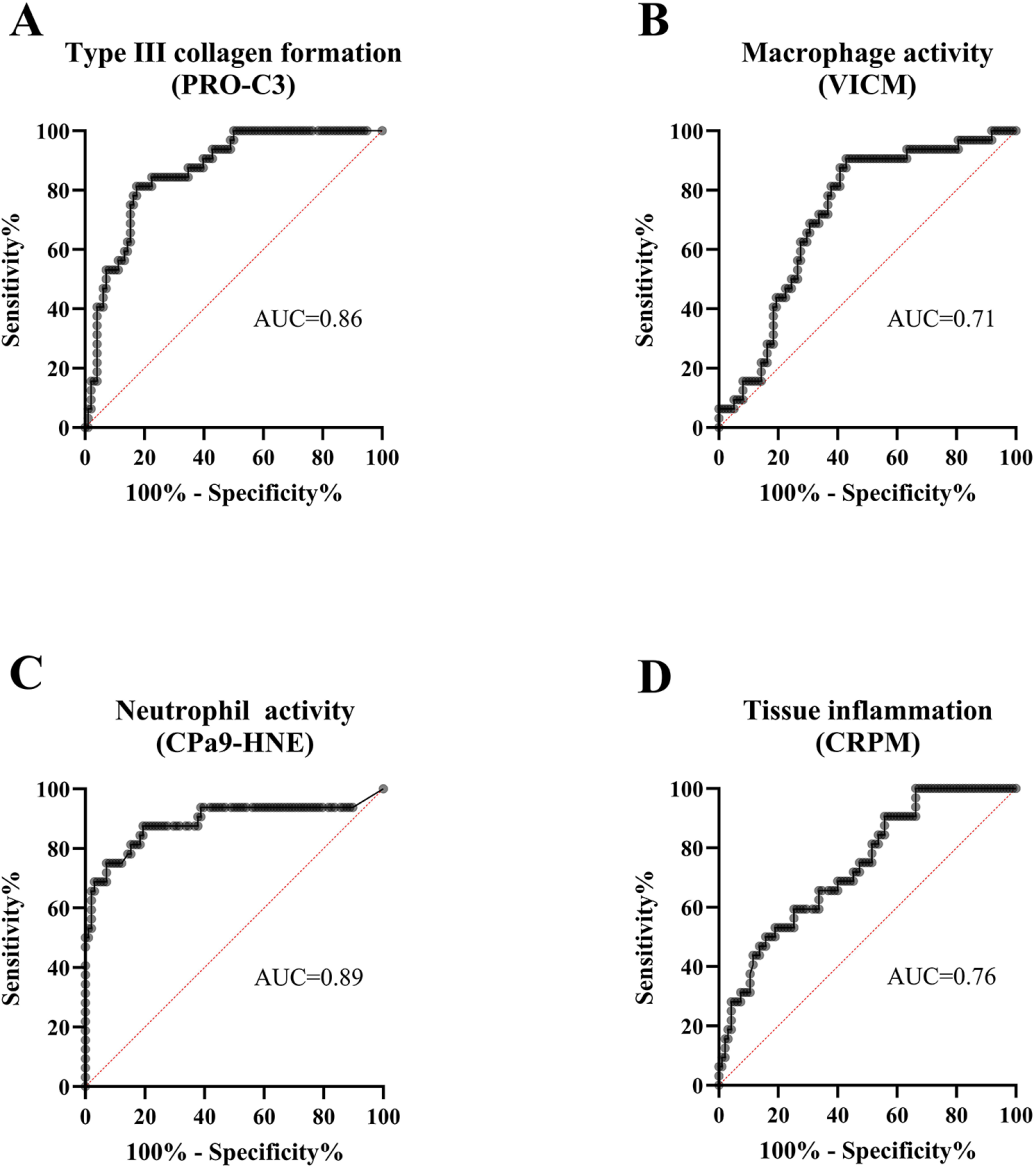
**A-D**. Discriminatory performance of PRO-C3, VICM, CPa9-HNE, and CRPM for separating PsA-flare from PsA without flare. The discrimination accuracy of each biomarker was analyzed using ROC curve analysis. PsA, Psoriatic Arthritis; ROC, Receiver Operating Characteristic

**Fig. 4:**
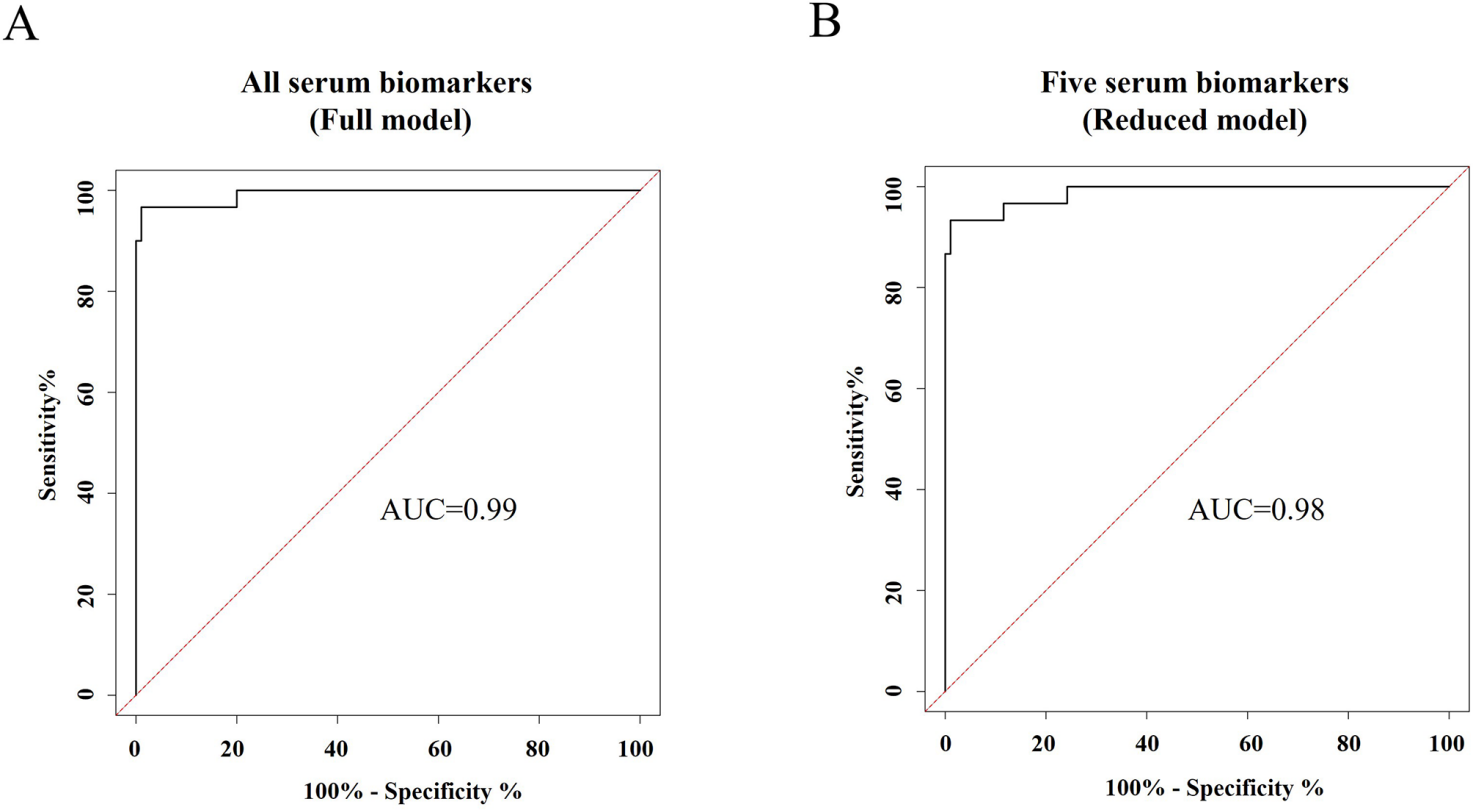
**A-B**. Discriminatory performance of combining the serum biomarkers for separating PsA-flare from PsA without flare. The five serum biomarkers include C3M, C4M, PRO-C3, PRO-C6, and CPa9-HNE. PsA, Psoriatic Arthritis; ROC, Receiver Operating Characteristic

Among all biomarkers measured in serum, which were evaluated by ROC curve analysis, PRO-C3, VICM, CPa9-HNE, and CRPM showed the good ability to separate PsA-flare from PsA without flare (Table 5; Fig. 3A-D). CPa9-HNE, reflecting neutrophil activity, demonstrated the highest ability for distinguishing patients with PsA-flare from those with PsA without flare with an AUC of 0.89 (95% CI (0.82–0.94), *p* < 0.0001, Fig. 3C). The fibrotic biomarker, PRO-C3, had an AUC of 0.86 (95% CI (0.79–0.92), *p* < 0.0001, Fig. 3A), and the systemic inflammation marker, CRPM, showed an AUC of 0.76 (95% CI (0.68–0.83), *p* < 0.0001, Fig. 3D). Lastly, the biomarker of macrophage activity, VICM, had the ability to separate PsA-flare from PsA with flare with an AUC of 0.71 (95% CI (0.62–0.79), *p* < 0.0001, Fig. 3B). To further investigate the association between serum levels of the 10 biomarkers with PsA-flare, multivariable regression analysis comparing marker levels between PsA-flare and PsA without flare provided an AUC of 0.99 (95% CI (0.98-1), Fig. 4A; Table 6). The reduced model of 5 markers provided an AUC of 0.98 (95% CI (0.97-1), Fig. 4B; Table 6).

**Table 5:** Results from the ROC curve analysis assessing the ability of each serum biomarker to discriminate PsA without flare from PsA-flare patients. PsA, Psoriatic Arthritis

Variables	AUC 95% CI	*p*-value	Criterion value	Sensitivity %	Specificity %
C1M	0.64 (0.55–0.72)	0.010	> 36.19	86.67	42.86
C3M	0.69 (0.60–0.77)	0.001	> 15.60	76.67	56.12
C4M	0.51 (0.42–0.60)	0.861	> 27.56	93.33	22.45
C6M	0.59 (0.50–0.68)	0.118	> 20.66	80.00	37.76
PRO-C3	0.86 (0.79–0.92)	< 0.0001	> 11.40	80.00	82.65
PRO-C4	0.66 (0.57–0.74)	< 0.004	> 211.23	80.00	51.02
PRO-C6	0.63 (0.54–0.71)	< 0.028	> 6.99	70.00	57.14
VICM	0.71 (0.62–0.79)	< 0.0001	> 6.99	90.00	57.14
CPa9-HNE	0.89 (0.82–0.94)	< 0.0001	> 203.20	76.67	92.86
CRPM	0.76 (0.68–0.83)	< 0.0001	> 9.63	63.33	74.74

**Table 6:** Multivariable analyses demonstrating independent association between serum biomarker levels and PsA-flare when compared to PsA without flare. PsA, Psoriatic Arthritis

	Full Model			Reduced Model			
	Odds Ratio	95% CI	*p*-value	Odds Ratio	95% CI	*p*-value	
C1M	0.97	(0.85, 1.10)	0.633				
C3M	1.70	(0.64, 4.53)	0.288	1.68	(1.08, 2.60)	0.020	
C4M	0.81	(0.59, 1.12)	0.201	0.80	(0.66, 0.98)	0.031	
C6M	0.90	(0.69, 1.16)	0.418				
PRO-C3	2.32	(1.33, 4.05)	0.003	2.27	(1.42, 3.63)	< 0.001	
PRO-C4	1.01	(0.99, 1.02)	0.599				
PRO-C6	0.59	(0.31, 1.13)	0.112	0.63	(0.44, 0.90)	0.011	
CPa9-HNE	1.05	(1.02, 1.10)	0.005	1.04	(1.02, 1.07)	< 0.001	
VICM	1.09	(0.98, 1.22)	0.097				
CRPM	1.58	(0.69, 3.64)	0.281				

**Fig. 5:**
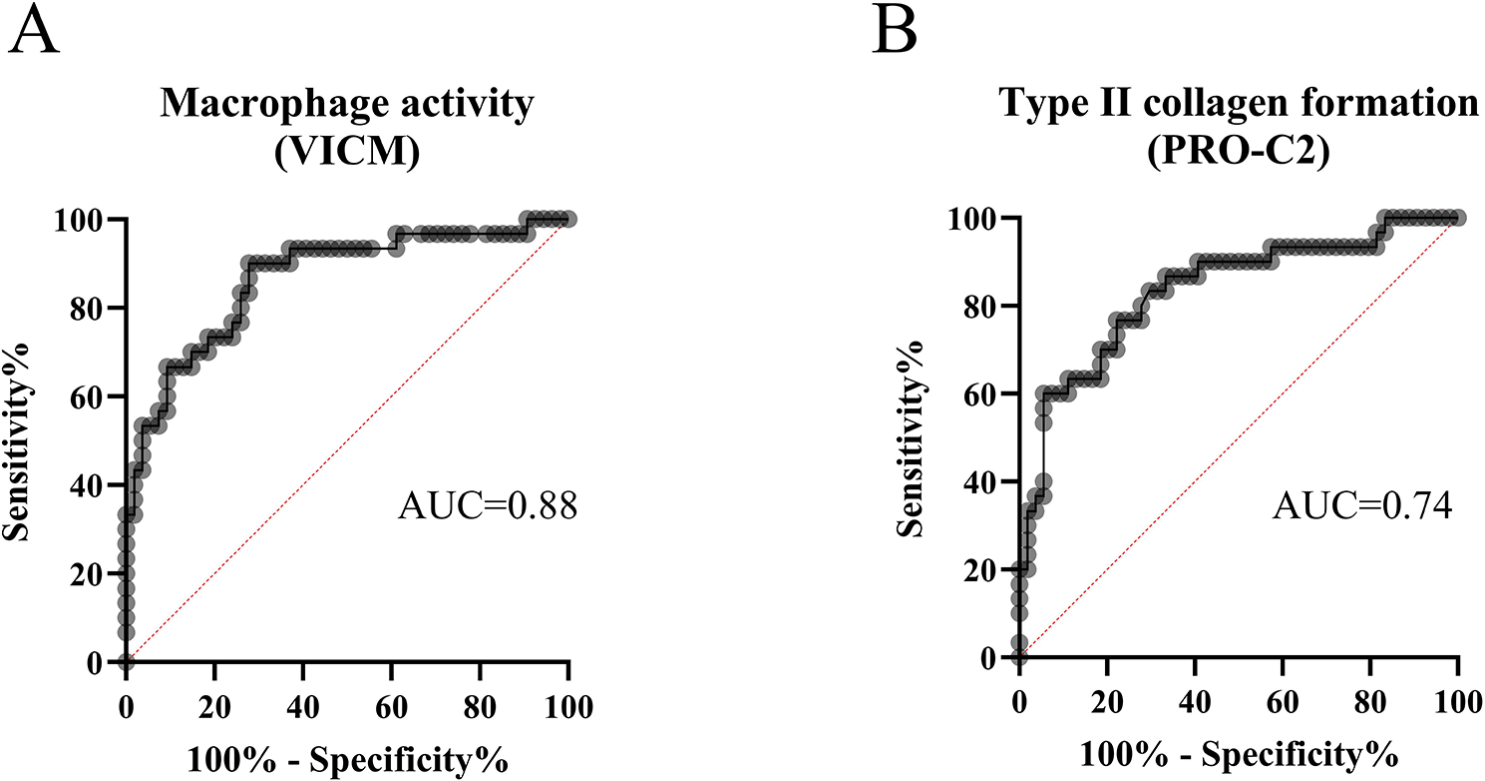
**A-B**. Discriminatory performance of VICM and PRO-C2 for separating OA from PsA-flare. PsA, Psoriatic Arthritis; OA, Osteoarthritis; ROC, Receiver Operating Characteristic

**Fig. 6:**
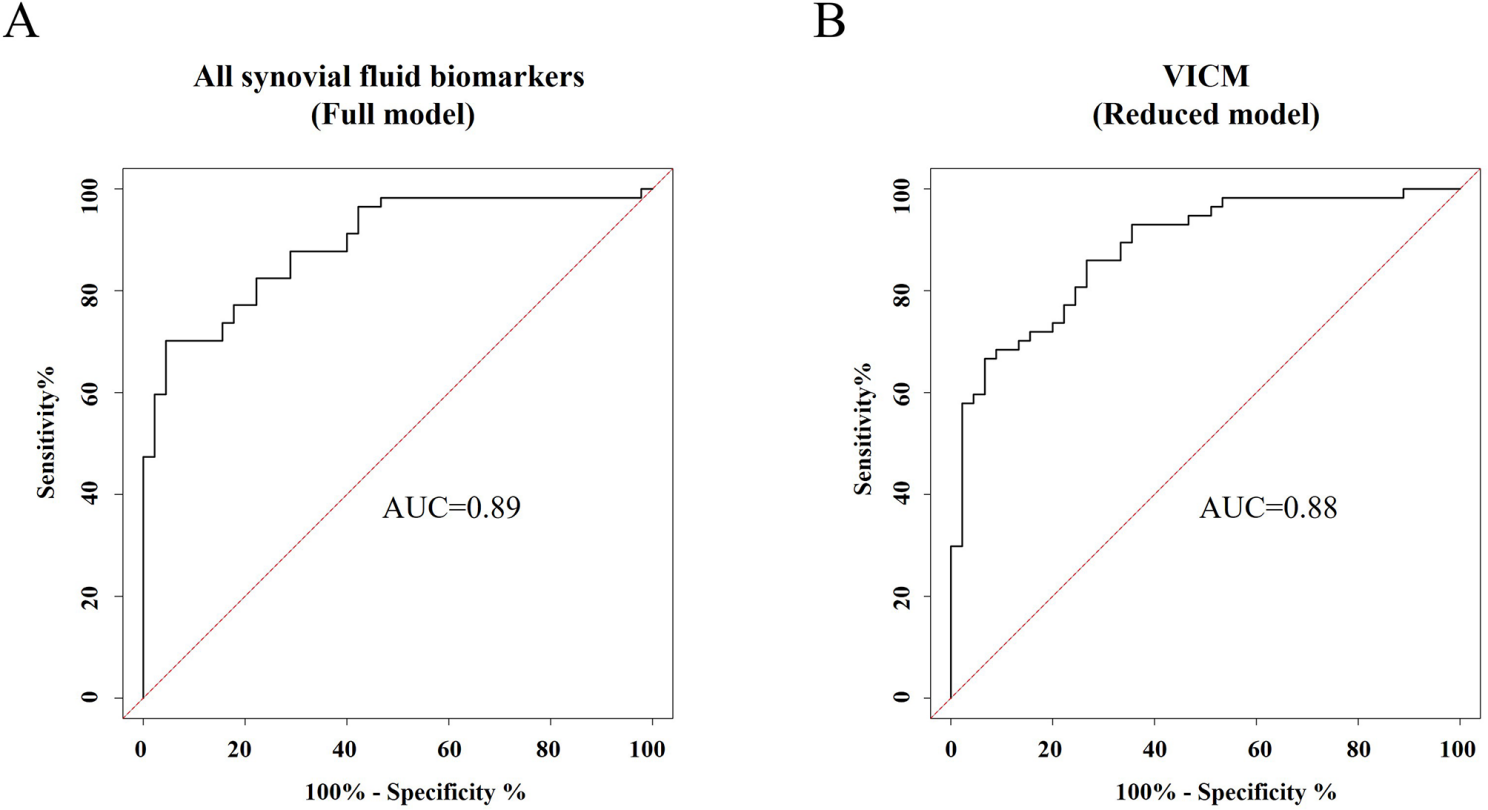
**A-B**. Discriminatory performance of synovial fluid biomarker panels distinguishing OA patients from PsA-flare. PsA, Psoriatic Arthritis; OA, Osteoarthritis; ROC, Receiver Operating Characteristic

In addition, the biomarkers, VICM, CPa9-HNE, FBN-C, PRO-C2 and ARGS, measured in SF from OA or PsA-flare were evaluated by ROC curve analysis (Table 7). Here, the macrophage activity marker, VICM, and type II collagen formation marker, PRO-C2, demonstrated best ability to discriminate between OA and PsA-flare. The AUCs were 0.88 (95% CI (0.80–0.93), *p* < 0.0001, Fig. 5A) and 0.74 (95% CI (0.65–0.82), *p* = 0.002, Fig. 5B), respectively. The multivariable analysis of all 5 biomarkers, adjusted for age, comparing PsA-flare to OA provided an AUC of 0.89 (95% CI (0.83–0.95), Fig. 6A; Table 8). The reduced model with VICM provided an AUC of 0.88 (95% CI (0.81–0.95), Fig. 6B; Table 8).

**Table 7:** Results from the ROC curve analysis assessing the ability of each synovial fluid biomarker to discriminate OA from PsA-flare patients. PsA, Psoriatic Arthritis; OA, Osteoarthritis

Variables	AUC 95% CI	*p*-value	Criterion value	Sensitivity %	Specificity %
VICM	0.88 (0.80–0.93)	< 0.0001	> 0.60	70.18	90.74
CPa9-HNE	0.51 (0.41–0.61	0.768	> 24.7	71.93	37.78
FBN-C	0.62 (0.53–0.71)	0.562	≤ 13060.9	57.89	64.81
ARGS	0.73 (0.50–0.68)	0.064	≤ 3024	61.40	94.44
PRO-C2	0.74 (0.65–0.82)	0.002	< 7.03	73.68	72.22

**Table 8:** Multivariable analyses adjusted for age demonstrating independent association between synovial fluid biomarker levels and PsA-flare when compared to OA. PsA, Psoriatic Arthritis; OA, Osteoarthritis

	Full Model			Reduced Model			
	Odds Ratio	95% CI	*p*-value	Odds Ratio	95% CI	*p*-value	
VICM	1.63	(1.03, 2.60)	0.039	1.76	(1.06, 2.93)	0.028	
CPa9-HNE	1.01	(0.97, 1.05)	0.548				
FBN-C	1.00	(1.00, 1.00)	0.760				
ARGS	1.00	(1.00, 1.00)	0.175				
PRO-C2	0.97	(0.94, 1.01)	0.145				
Age	0.91	(0.86, 0.96)	< 0.001	0.90	(0.86, 0.94)	< 0.001	

### Association between the biomarkers and clinical parameters

An association analysis was performed between the biomarkers measured in serum and clinical parameters and routine laboratory measures of PsA without flare patients. A positive correlation was identified between CPa9-HNE and hsCRP (*r* = 0.286, *p* = 0.010). Furthermore, in the PsA-flare patients, a positive correlation was observed between CRPM and hsCRP (*r* = 0.484, *p* = 0.026, Supplementary Table 2).

## Discussion

In a first of its kind study addressing the unmet need for biomarkers in the field, the study aimed to investigate protease-mediated markers of inflammation and tissue remodeling as biomarkers of flares in PsA patients. These biomarkers were measured in serum and SF from PsA patients experiencing flares defined as an acutely swollen and tender joint requiring intra-articular injection with corticosteroids. The serum levels were compared to controls and PsA patients without flares (who might have active disease, but were not experiencing flares that is any change in disease activity), and SF levels were compared to OA, a commonly used less inflammatory arthritic control.

In serum, levels of PRO-C3 and C3M, which are related to formation and degradation of the interstitial matrix, were found to be elevated in PsA with flares compared to controls and PsA without flare. The remodeling marker of the basement membrane, PRO-C4, was elevated in PsA-flare compared to PsA without flare. The inflammation and immune cell activity related markers, CRPM, VICM, and CPa9-HNE were highly elevated in PsA-flare patients compared to both controls and PsA without flare patients. Furthermore, PRO-C3 and CPa9-HNE showed best discriminatory performance for separating PsA patients with flares from PsA without flare patients. In the multivariable analysis, a combination of the biomarkers displayed superior value as a serum biomarker panel to distinguish between PsA-flare from PsA without flares, thus as a tool for monitoring flares and by extension, elevated disease activity in PsA patients.

A likely explanation for CPa9-HNE being one of the best markers to discriminate elevated disease activity in PsA patients may be that neutrophils are playing an important role in psoriatic diseases. Neutrophils can release a program of the neutrophil extracellular trap formation (NETosis) which may take part in the pathogenesis of psoriasis and PsA [10–12]. Recently, a NETosis marker has been investigated as a potential disease activity biomarker in PsA, the myeloperoxidase–DNA (MPO–DNA) complex, one of the neutrophil extracellular trap components. In this study, the level of MPO–DNA complex was markedly elevated in serum samples from patients with PsA and psoriasis and further shown to positively correlate with psoriatic disease burden [12]. Another research group investigated immature neutrophils as disease activity biomarker in PsA. It was shown that the immature neutrophil score was elevated in PsA patients compared to controls and significantly correlated with disease activity based on clinical parameters (ASDAS, SPARCC, PASI, ESR, CRP, enthesitis, dactylitis) [13]. Lastly, serum calprotectin has been explored as biomarkers of disease activity in PsA patients and found to be significantly elevated in patients with psoriasis and PsA compared with controls, and further positively correlated with psoriasis area and severity PASI, DAPSA, TJC, and SJC [14]. Overall, evidence from previous findings indicates that neutrophils play a key role in psoriatic disease, thus this may explain why CPa9-HNE levels are particularly elevated in PsA patients with flares and may be a useful biomarker to monitor elevated disease activity. Additionally, PRO-C3 also showed to be one of the best markers to discriminate elevated disease activity in PsA patients. PRO-C3 measures type III collagen formation and has been reported to be a marker of fibrogenesis [15]. In previous studies, PRO-C3 showed to be connected with the activation of dermal and lung fibroblasts and upregulated in patients with systemic sclerosis (SSc), associated with progressive disease [16, 17]. Hence, it can be questioned whether PRO-C3 is upregulated during flares in this study as a result of progressive fibrosis or because of an increased repair mechanism that is activated to counteract the upregulated joint tissue degradation mirrored by the elevated collagen degradation, in particular the type III degradation (C3M).

Besides assessing the elevated disease activity systemically in the serum from PsA patients, it was further investigated whether the elevated disease activity could be observed in the local joint microenvironment using SF. Two panels of biomarkers were chosen in the SF samples, where one panel consisted of markers reflecting inflammation and the other reflecting cartilage remodeling. It was hypothesized that the inflammatory biomarkers would be elevated in PsA known to be an inflammatory disease, while the cartilage degradation markers would be elevated in OA, known to be a degenerative disease, where cartilage degradation is a hallmark. Interestingly, one of the inflammation markers reflecting macrophage activity, VICM, showed to be elevated in PsA-flare, whereas the marker of neutrophil activity, CPa9-HNE, was equally expressed in PsA-flare compared to OA patients. Neutrophils are known to play an important role in the PsA pathology at the site of local inflammation where enhanced IL-23 production and Th17 activation trigger neutrophil infiltration and increased concentrations of calprotectin [18]. Therefore, it is notable that in this study the neutrophil activity levels are not significantly elevated in the PsA-flare patients compared to OA. On the other hand, the macrophage activity was highly elevated in PsA-flare patients compared to OA. These elevated levels may reflect the synovial tissue macrophages, which are recognized as pro-inflammatory cells previously shown to have a key role in rheumatoid arthritis by producing TNF, in turn driving chronic pathology [19]. SF macrophages have also been recently shown to play key roles in joint inflammation [20]. Altogether, these results may suggest distinct functions of neutrophils and macrophages during PsA-flare, where macrophages may be driving the disease activity both locally and systemically.

Furthermore, among the markers of cartilage remodeling, PRO-C2 reflecting type II collagen formation, was the only marker being elevated in OA patients compared to PsA-flare. The elevated levels of type II collagen formation may reflect an imbalance between the formation and degradation processes of type II collagen. While no biomarkers were available for the assessment of type II collagen degradation in SF, the elevated type II collagen levels may be a result of an imbalance in the tissue homeostasis, where elevated degenerative processes compensate with enhanced formation of type II collagen to maintain the tissue homeostasis. Collectively, these findings show that there are overlaps in the pathology of OA and PsA patients, where some OA patients present with inflammatory symptoms, whereas PsA patients additionally have increased cartilage degradation. Hence, these results may highlight the difficulty in telling the two types of arthritis apart in the clinic.

The matched serum and synovial fluid samples from the PsA-flare patients were analyzed to investigate potential marker differences in the circulation versus the local joint microenvironment. The biomarkers CPa9-HNE and VICM were analyzed since these biomarker assays are technically validated for measurement in both serum and SF matrix. Results from the analysis showed that the mean levels of CPa9-HNE and VICM were significantly elevated in serum compared to synovial fluid. The higher levels observed in the serum compared to SF may indicate higher systemic macrophage and neutrophil activity that is not confined locally to the knee joints. Notably, no correlation was observed between the systemic and local levels of the PsA-flare patients, which may indicate the heterogeneity characterizing the PsA disease.

Interestingly, in this study no significant difference was observed in biomarkers levels between controls and PsA without flare patients. One possible explanation for this may be that the controls are self-reported healthy donors with limited clinical data available; thus, some donors may have underlying conditions affecting systemic marker levels. In one study, where CPa9-HNE was assessed in serum from healthy donors, the median value of CPa9-HNE was 30 ng/mL where patients with ulcerative colitis or Crohn’s disease had levels all above 100 ng/mL [21]. In this work, the controls presented a median value of 125 ng/mL, which is a level more similar to the patients presenting ulcerative colitis and Crohn’s disease. Another explanation for the similar biomarker levels observed between controls and PsA without flare may be that these biomarkers are end products of elevated protease activity as a result of extensive tissue inflammation, which may be significantly lower in the PsA patients without flare. The PsA without flare patients have only been diagnosed with PsA disease within 1.9 years in average compared to PsA-flare patients who have had the disease for 11.5 years in average. Interestingly, when only observing the clinical parameters of the PsA without flare patients and PsA-flare patients, the hsCRP levels were similar between the two groups, although the PsA without flare patients have significantly higher PASI and DAPSA scores. The tender joint count and active joint criteria were significantly higher in PsA without flare compared to PsA-flare. PsA-flares were defined by the presence of an acutely swollen and tender knee joint whereas PsA without flare patients did not have such acute changes in disease activity. None of the PsA without flare patients were receiving t-DMARD, while 40% of PsA-flare patients were treated with t-DMARD. The wide range in joint counts seen for the PsA patients are likely due to the effect of treatment and the criteria for study entry. However, highly elevated levels of the inflammation markers including the fibrotic marker were found in serum from the PsA-flare patients when compared to PsA without flare and controls. Based on these data, the ability of hsCRP as a good marker to monitor disease activity may be questioned. Furthermore, in the PsA without flare patients, marginally significant correlation was observed between hsCRP and CPa9-HNE.

Limitations of this study include the cross-sectional design and the fact that the number of serum samples from PsA-flare group (*n* = 30) was three times lesser than the controls (*n* = 99) and PsA without flare group (*n* = 98). Given the unmet need for disease activity and flare biomarkers in the management of PsA, there is an immediate need to conduct longitudinal internal and external validation studies with an expanded cohort of patients to confirm the findings of this study.

## Conclusion

In conclusion, a biomarker panel of ECM turnover and systemic inflammation was evaluated in a large sample of psoriatic disease patients. Serum biomarkers of tissue remodeling and inflammation were elevated in PsA patients with flares compared to PsA patients without flares. Biomarkers of neutrophil activity and fibrosis showed best discriminatory performance for separating PsA patients with flares from PsA without flare patients and may serve as potential biomarkers of disease activity associated with proteolytic activity in the joints. A multiparametric serum biomarker panel was also identified as potential biomarkers of PsA-flare. Longitudinal studies need to be conducted to better define the relationship between these biomarkers and PsA disease activity, including flares. In the future, such a marker or marker panel may be used to evaluate whether there is a need for alternative therapeutic management and/or preventive therapeutic intervention prior to a flare.

### Electronic supplementary material

Below is the link to the electronic supplementary material.

Supplementary Material 1

## Data Availability

No datasets were generated or analysed during the current study.

## Abbreviations

AJC: Active (swollen or tender) joint criteria
ARGS: ADAMTS-degraded fragment of aggrecan
ASDAS: AS Disease Activity Score based on C-Reactive Protein
AUC: Area under the curve
BMI: Body mass index
C1M: MMP-degraded type I collagen
C3M: MMP-degraded type III collagen
C4M: MMP-degraded type IV collagen
C6M: MMP-degraded type VI collagen
CPa9-HNE: HNE-mediated degradation of calprotectin
CRP: C-reactive protein
CRPM: C-reactive protein metabolite
c-DMARD: Conventional disease-modifying antirheumatic drug
ESR: Erythrocyte sedimentation rate
DAPSA: Disease activity index for PsA
ECM: Extracellular matrix
FBN-C: C-terminal of fibronectin
hsCRP: High-sensitive c-reactive protein
MMPs: Matrix metalloproteinases
OA: Osteoarthritis
PRO-C2: Pro-peptide of type II collagen
PRO-C3: Pro-peptide of type III collagen
PRO-C4: Type IV 7 S domain collagen
PRO-C6: Type VI alpha-3 chain collagen
PsA: Psoriatic arthritis
ROC: Receiver Operating Characteristic
SF: Synovial fluid
SJC: Swollen joint count
SPARCC: Spondyloarthritis Research Consortium of Canada
t-DMARD: Targeted disease-modifying anti-rheumatic drug
TICOPA: TIght Control Of Psoriatic Arthritis
VICM: Citrullinated and MMP-degraded vimentin
