# Supplementary material for: Investigating protease-mediated peptides of inflammation and tissue remodeling as biomarkers associated with flares in psoriatic arthritis
Source: Arthritis Res Ther. 2024 May 27;26:107. doi: 10.1186/s13075-024-03332-7 (PMC11129460; doi:10.1186/s13075-024-03332-7)
Supplement: Supplementary file 1 — Supplementary Material 1 [file 13075_2024_3332_MOESM1_ESM.docx]

**Supplementary Figure 1 A-B. Biomarkers measures in matched serum and synovial fluid samples from PsA-flare patients.**

**Supplementary Table 1. Correlation between biomarker measures in serum and synovial fluid from PsA-flare patients**

| PsA-flare | | |
| --- | --- | --- |
| Biomarkers | VICM  serum | VICM  synovial fluid |
| CPa9-HNE  serum | Ns | Ns |
| CPa9-HNE  Synovial fluid | Ns | Ns |

Ns, non-significant.

**Supplementary Table 2. Correlations biomarkers and clinical parameters of the PsA without flare and PsA-flare patients.**

| PsA without flare | | | | |
| --- | --- | --- | --- | --- |
| Biomarkers | PRO-C3 | VICM | CPa9-HNE | CRPM |
| PsA duration | Ns | Ns | Ns | Ns |
| PASI | Ns | Ns | Ns | Ns |
| SJC | Ns | Ns | Ns | Ns |
| TJC | Ns | Ns | Ns | Ns |
| AJC | Ns | Ns | Ns | Ns |
| Axial | Ns | Ns | Ns | Ns |
| hsCRP | Ns | Ns | r=0.286 *p=*0.010 | Ns |
| NSAID | Ns | Ns | Ns | Ns |
| c-DMARD | Ns | Ns | Ns | Ns |
| t-DMARD | Ns | Ns | Ns | Ns |
| DAPSA | Ns | Ns | Ns | Ns |
| PsA-flare | | | | |
| Biomarkers | PRO-C3 | VICM | CPa9-HNE | CRPM |
| PsA duration | Ns | Ns | Ns | Ns |
| PASI | Ns | Ns | Ns | Ns |
| SJC | Ns | Ns | Ns | Ns |
| TJC | Ns | Ns | Ns | Ns |
| AJC | Ns | Ns | Ns | Ns |
| Axial | Ns | Ns | Ns | Ns |
| hsCRP | Ns | Ns | Ns | r=0.484 *p=*0.026 |
| NSAID | Ns | Ns | Ns | Ns |
| c-DMARD | Ns | Ns | Ns | Ns |
| t-DMARD | Ns | Ns | Ns | Ns |
| DAPSA | Ns | Ns | Ns | Ns |

Ns, non-significant; PsA, psoriatic arthritis; PASI, psoriasis area and severity index; SJC, Swollen joint count; TJC, Tender joint count; AJC, Active joint criteria; hsCRP, high-sensitive c-reactive protein; NSAID, Nonsteroidal anti-inflammatory drug; c-DMARD, conventional disease-modifying antirheumatic drug; t-DMARD, targeted disease-modifying anti-rheumatic drug; DAPSA, Disease activity in PsA.
